# The Critical Role of Soil Ecological Stoichiometric Ratios: How Does Reforestation Improve Soil Nitrogen and Phosphorus Availability?

**DOI:** 10.3390/plants13162320

**Published:** 2024-08-20

**Authors:** Zhixuan Chen, Xia Xu, Yongli Wen, Man Cheng, Xiao Wang

**Affiliations:** 1 Institute of Loess Plateau, Shanxi University, Taiyuan 030006, China; dxx994996@163.com (Z.C.); 15235710610@163.com (X.X.); ylwen@sxu.edu.cn (Y.W.); 2 School of Environmental and Resource Sciences, Shanxi University, Taiyuan 030006, China; 3 Key Laboratory of Forest Ecology and Environment of National Forestry and Grassland Administration, Ecology and Nature Conservation Institute, Chinese Academy of Forestry, Beijing 100091, China

**Keywords:** soil nutrients, ecological stoichiometry, restoration time, vegetation type, soil depth

## Abstract

The ecological stoichiometric characteristics of soil elements have greatly enhanced our understanding of the circulation of soil nutrients. However, there is limited knowledge regarding the alteration of carbon, nitrogen, and phosphorus stoichiometric ratios in deep soil after afforestation. To examine the variations in stoichiometric ratios of soil elements with different vegetation types, restoration times, and soil depths, we collected soil samples from grassland, *Caragana korshinskii* shrubland, and *Picea asperata* forestland at different stand ages (10a, 25a, and 40a) in Xining City, which is located on the Loess Plateau. Our results showed that, at 25a, the carbon-to-nitrogen (C:N) and carbon-to-phosphorus (C:P) ratios were significantly higher in the grassland soil than under other vegetation types, whereas the nitrogen-to-phosphorus (N:P) ratio had no significant difference among the three vegetation types. At 40a, the ratios of soil C:N, C:P, and N:P in the shrubland were the highest. With the increasing of the restoration time, the ratios of soil C:N, C:P, and N:P in grassland with 25a became higher than for 40a or 10a. The ratios in the shrubland were highest at 40a, followed by 25a and then 10a, while the ratios in the forestland showed no significant difference. At 40a, the soil C:N, C:P, and N:P ratios of shrubland were highest at the soil depth of 40–100 cm. The soil C:N, C:P, and N:P ratios showed positive correlations with soil ammonium nitrogen and nitrate nitrogen, and the soil N:P ratios showed a negative correlation with soil available phosphorus. Plant diversity significantly influenced the soil stoichiometric ratio of the upper soil layer. In the upper soil layer (0–40 cm), species richness showed a positive correlation with soil C:N, C:P, and N:P ratios, and the Margalef index exhibited a positive correlation with soil C:N and C:P ratios. The results of this study indicate that the stoichiometric ratio and nutrient availability of *Caragana korshinskii* shrubland were the highest over time. Therefore, these findings can be served as a valuable reference for local revegetation and ecological restoration.

## 1. Introduction

Ecological stoichiometry is the science of studying the cycling of chemical elements (carbon, nitrogen, phosphorus, etc.) and the energy balance in ecosystems [1]. The study of the stoichiometric characteristics of soil C, N, and P can not only reveal the interactions and balance among C, N, and P but also provide information on the availability of nutrients, which can significantly improve the understanding of the development of soil ecosystems. For instance, the ratio of soil carbon to nitrogen (C:N) can be considered as a predictor of nitrogen mineralization potentiality, the ratio of carbon to phosphorus (C:P) shows soil phosphorus availability, and the ratio of nitrogen to phosphorus (N:P) establishes a threshold for nutrient limitation [2]. The soil ecological stoichiometric ratios also determine the growth of plants and soil microbes, which dominate the changes of community structure and function [3]. Previous studies show that non-leguminous plants are usually suppressed when the soil N:P ratio < 10 [4]; however, leguminous plants can enhance their ability for nodule nitrogen fixation by adjusting the allocation of carbon sources between roots and nodules, thereby providing timely supplementation of required nitrogen sources for growth and development [5]. On the other hand, a soil C:N ratio of 10 promotes the growth of K-strategy fungi, whereas a soil C:N ratio of 4 promotes the proliferation of r-strategist bacteria, which are limited to utilizing simple carbon sources in the soil [6]. The different soil microbial communities have varied influence on the accumulation of soil carbon and nitrogen [6]. Therefore, soil ecological stoichiometry could provide important insights for understanding nutrient cycling, the equilibrium and coupling of the main constituents, and ecosystem dynamics.

Afforestation is a crucial strategy for enhancing and rehabilitating degraded land, sequestering carbon, regulating the regional hydrological cycle, improving water quality, etc. [7,8]. In addition, afforestation generally alters the composition and structure of plant communities, as well as the composition of understory species. It also affects the accumulation and distribution of soil nutrients such as carbon, nitrogen, and phosphorus through litter input and root systems [9,10,11,12]. Researchers have conducted numerous studies on the soil ecological stoichiometric characteristics under various levels of vegetation restoration, encompassing diverse durations of restoration [13], varying vegetation types [14], different altitudes [15], distinct seasons [16], and other pertinent factors. The age of a forest, known as the stand age, is a crucial factor in reforestation, which can impact soil nutrient distribution by altering fractions of forest materials [17]. For example, Hume et al. [18] found that the ratios of soil C:N, C:P, and N:P in the 0–30 cm soil layer increased with the lengthening of restoration time after fire disturbance in the boreal forest of central Canada. Lucas-Borja et al. [19] conducted a study on the secondary succession of temperate pine forests in central-eastern Spain and revealed that the soil C:N ratio in the 0–10 cm soil layer decreases during ecosystem succession (1–120a). Zethof et al. [20] revealed that the soil C:N ratio increased with the duration of afforestation in the 0–10 cm soil layer in southeastern Spain. Niu et al. [21] reported that the soil C:P and N:P ratios initially increased (0–50a) and then decreased (50a–65a) in the 0–20 cm soil layer of *Robinia pseudoacacia* forestland, which is located in the middle of the loess hilly area of China. Cao et al. [22] demonstrated that the soil C:N and C:P ratios increased with afforestation ages (22a–42a), while the soil N:P ratios first increased (22a–32a) and then decreased (32a–42a) in the 0–40 cm soil layer of *Picea asperata*, which is located in the eastern Tibet Plateau of China. Xu et al. [23] indicated that the soil C:P ratios decreased initially (3a–6a) and then increased (6a–13a) in the 0–20 cm soil layer of *Eucalyptus grandis x urophylla* forestland in South China subtropical areas. These studies have shown that the effect of afforestation on the ecological stoichiometric ratio of soil C, N, and P is uncertain and influenced by various factors such as temporal and spatial variations. Furthermore, most of the previous studies have primarily focused on the upper soil layer rather than the deep soil layer. However, the cycling of elements in deep soil is closely related to the long-term development of plant–soil systems. Therefore, it is imperative to study changes in the stoichiometric ratios of soil C, N, and P in deep soil layers under different afforestation ages and vegetation types, especially in ecologically fragile areas.

Xining City, located in the western part of the Loess Plateau, is a typical ecologically fragile area. In order to improve the eco-environment there, a reforestation project was conducted in the north and south mountains sequentially in 1981, 1996, and 2010, increasing the forest cover rate from 7.2% to 79.0%, thus significantly improving air quality and the human habitat [24]. Currently, the reforestation project in the north and south hills of Xining City has formed a pattern of multiple vegetation types. In this study, we chose the *Picea asperata* pure forestland, *Caragana korshinskii* shrubland, and grassland reforested for 40a, 25a, and 10a as the research object. We hypothesized that (i) the soil C:N, C:P, and N:P ratios of different vegetation types decrease in the order of *Picea asperata* pure forestland, *Caragana korshinskii* shrubland, and grassland; (ii) the C:N, C:P, and N:P ratios of the deep soil layer would increase with the development of reforestation; and (iii) with the increase of soil C:N, C:P, and N:P ratios, the availability of soil nitrogen and phosphorus would increase, as those ratios were affected by plant diversity. The objective of this study is to reveal the impact of shrub and arbor reforestation on the soil stoichiometric ratios (0–100 cm soil layer) in the typical alpine cold arid region of the Loess Plateau.

## 2. Results

### 2.1. Effects of Reforestation Age, Vegetation Type, and Soil Depth on Soil Carbon, Nitrogen, Phosphorus, and Soil Stoichiometric Ratios

We conducted a three-way ANOVA on the influence of reforestation time, vegetation type, and soil depth on the soil carbon, nitrogen, phosphorus, and soil stoichiometric ratios. The ANOVA results showed that SOC, STN, SNN, C:N, C:P, and N:P differed significantly at different levels in response to a single factor of reforestation time, vegetation type, and soil depth, while STP and SAN were only affected by reforestation time (Table 1). The interaction between reforestation time and vegetation type had significant effects on SOC, STN, SAP, SAN, SNN, C:N, C:P, and N:P. Furthermore, the interaction between reforestation time and soil depth had significant effects on SOC, STN, C:P, and N:P.

### 2.2. The Distribution of Soil C:N Stoichiometric Ratio

**Figure 1:**
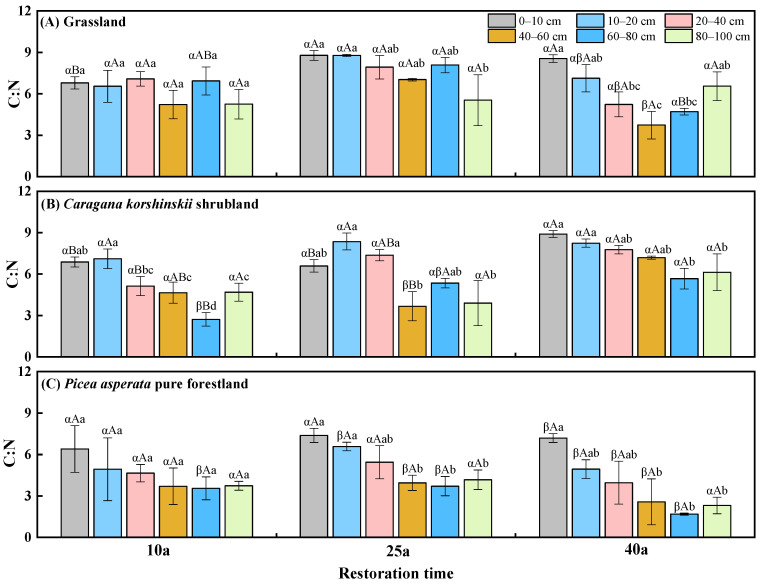
Soil C:N under different vegetation types with the development of reforestation. Greek letters indicate significant differences among different vegetation types with the same reforestation time (*p* < 0.05), capital letters indicate significant differences among different reforestation time under the same vegetation types (*p* < 0.05), and lowercase letters indicate significant differences among different soil depths in the same year (*p* < 0.05).

According to Figure 1, apart from the 60–80 cm soil layer, there was no significant difference in the soil C:N ratio under different vegetation types after 10a of reforestation. When the reforestation time came to 25a, the soil C:N ratio of the 10–20 cm, 40–60 cm, and 60–80 cm soil layers was 33.3–117.8% higher in the grassland than that in *Picea asperata* pure forestland. When the reforestation time came to 40a, the soil C:N ratio was the highest in shrubland, except for the 80–100 cm soil layer, and was 23.8–237.5% higher than that in the *Picea asperata* pure forestland. The soil C:N ratio of the 0–10 cm soil layer was significantly higher in the 40a and 25a grassland than in the 10a grassland by 25.9% and 29.3%, respectively, and the soil C:N ratio of the 60–80 cm soil layer was significantly lower in the 40a grassland than in the 25a grassland, by 41.8%. In the shrubland, the soil C:N ratio of the 0–10 cm soil layer was much higher at 40a than 25a and 10a by 35.1% and 29.3%, respectively. In the *Picea asperata* pure forestland, the soil C:N ratio did not change significantly with reforestation time. Apart from the grassland and forestland at 10a, the soil C:N ratio basically decreased with soil depth.

### 2.3. The Distribution of Soil C:P Stoichiometric Ratio

**Figure 2:**
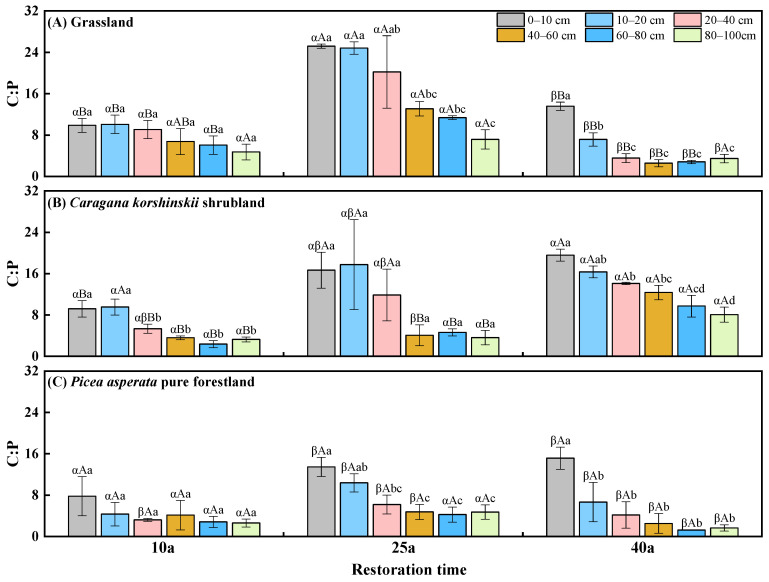
Soil C:P under different vegetation types with the development of reforestation. Greek letters indicate significant differences among different vegetation types with the same reforestation time (*p* < 0.05), capital letters indicate significant differences among different reforestation time under the same vegetation types (*p* < 0.05), and lowercase letters indicate significant differences among different soil depths in the same year (*p* < 0.05).

In the 10a reforestation, there was no significant difference in the soil C:P ratios among the different vegetation types; at 25a of reforestation, soil C:P ratios in the 0–40 cm soil layer were highest in the grassland, followed by shrubland, and the ratios were lowest in the forestland; at 40a of reforestation, the soil C:P ratios in the 0–100 cm soil layer were significantly higher in the shrubland compared to other vegetation types, showing an increase from 29.3% to 695.9% (Figure 2). The soil C:P ratio of grassland first increased (10a–25a) and then decreased (25a–40a) with reforestation. The soil C:P ratio at 25a was 85.5–857.0% higher than at 10a and 40a in the grassland, except for the 80–100 cm soil layer. The soil C:P ratios in shrubland showed an overall increasing trend with reforestation time, whereas there was no significant difference in forestland with reforestation time. Apart from the 10a grassland and forestland as well as 25a shrubland, the soil C:P basically decreased with increased soil depth.

### 2.4. The Distribution of Soil N:P Stoichiometric Ratio

**Figure 3:**
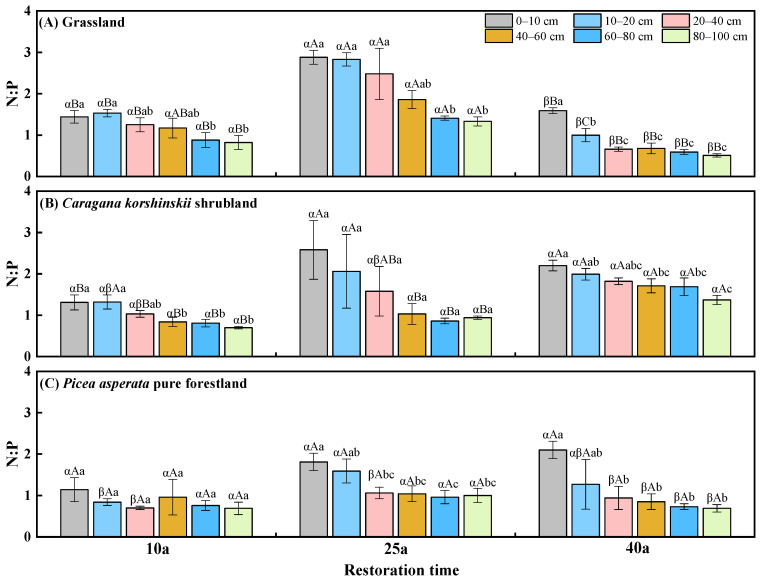
Soil N:P under different vegetation types with the development of reforestation. Greek letters indicate significant differences among different vegetation types with the same reforestation time (*p* < 0.05), capital letters indicate significant differences among different reforestation time under the same vegetation types (*p* < 0.05), and lowercase letters indicate significant differences among different soil depths in the same year (*p* < 0.05).

At 10a of reforestation, the soil N:P ratio of the 10–20 cm and 20–40 cm soil layers reached the highest point in the grassland and came to lowest in the *Picea asperata* pure forestland (Figure 3). At 25a of reforestation, the N:P ratios in the 0–100 cm soil layer were not significantly different among the vegetation types. At 40a of reforestation, except for the 0–10 cm soil layer, the N:P ratio in other soil layers of the *Caragana korshinskii* shrubland was 56.9–184.4% higher than that in the grassland and *Picea asperata* pure forestland. The soil N:P ratio showed an increasing trend followed by a decreasing trend in the grassland with the increase of the reforestation time. Specifically, the N:P ratio at 25a was significantly higher than that at 40a and 10a in the grasslands, showing an increase ranging from 59.7% to 278.6%. The soil N:P increased in the *Caragana korshinskii* shrubland with reforestation time; meanwhile, it showed no significant difference in the *Picea asperata* pure forestland with reforestation time. The soil N:P ratio generally decreased with soil depth, except for the 25a shrubland and 10a forestland.

### 2.5. The Relationship between Soil C, N, and P Stoichiometric Ratios and Soil Physico-Chemical Properties across Soil Profile with Reforestation

As can be seen from Table 2, the soil C:N, C:P, and N:P ratios were all significantly and positively correlated with SNN and P_S_ (*p* < 0.001). Additionally, they showed significant positive correlations with SAN (*p* < 0.05) and highly significant negative correlations with BD (*p* < 0.001). Moreover, they displayed significant negative correlations with soil pH (*p* < 0.05).

**Figure 4:**
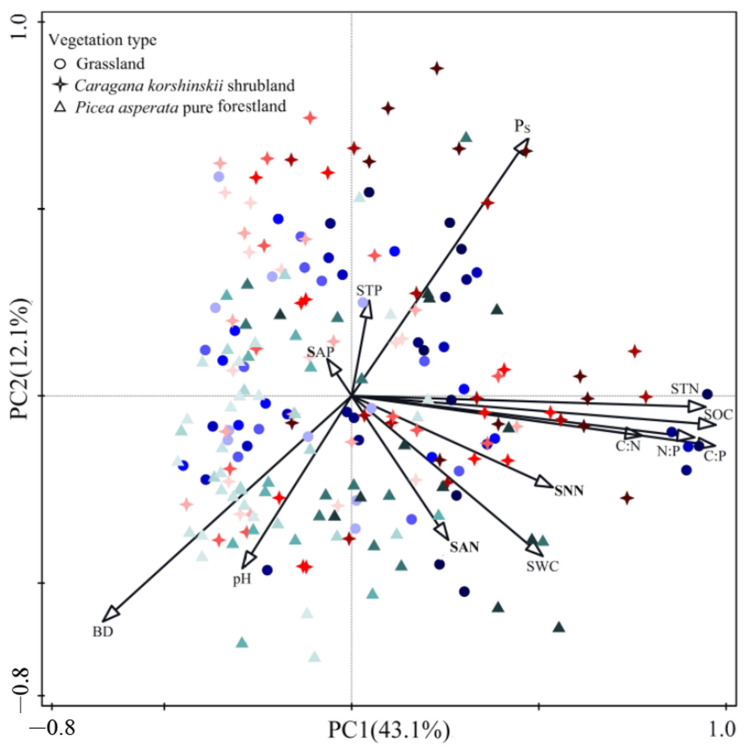
Principal component analysis (PCA) of soil stoichiometric ratios and physico-chemical properties. C:N, soil carbon–nitrogen ratio; C:P, soil carbon–phosphorus ratio; N:P, soil nitrogen–phosphorus ratio; pH, soil acidity–alkalinity; SOC, soil organic carbon; STN, soil total nitrogen; STP, soil total phosphorus; SAP, soil available phosphorus; SAN, soil ammonium nitrogen; SNN, soil nitrate nitrogen; SWC, soil water concentrations; BD, soil bulk density; Ps, soil total porosity; the color from dark to light indicates that the soil layer is from shallow to deep (0–100 cm).

Principal component analysis (PCA) explained 43.1% of the total variance on axis 1 and 12.1% of the total variance on axis 2, reaching a total explanation of 55.2% (Figure 4). The results of the principal component analysis (PCA) showed that the first principal component was mainly positively correlated with SOC, STN, SWC, SNN, C:N ratio, C:P ratio, and N:P ratio. The second principal component was mainly positively correlated with soil Ps, STP, and SAP and negatively correlated with BD, pH, and SAN. Meanwhile, the principal component analysis (PCA) also showed that soil in the 0–10 cm and 10–20 cm soil layers of the grassland, *Caragana korshinskii* shrubland, and *Picea asperata* pure forestland were essentially distributed in the first and fourth quadrants, and the soil in the 60–80 cm and 80–100 cm soil layers was essentially concentrated in the second and third quadrants. The scattergram clearly delineated higher soil C:N, C:P, and N:P ratios in the 0–10 cm and 10–20 cm soil layers under the three vegetation types, which could be considered as carbon-rich and nitrogen-rich soil layers, whereas there was a lower soil C:N, C:P, and N:P ratio in the 60–80 cm and 80–100 cm soil layers, which, therefore, could be considered as the oligo-carbon and oligo-nitrogen soil layers.

**Figure 5:**
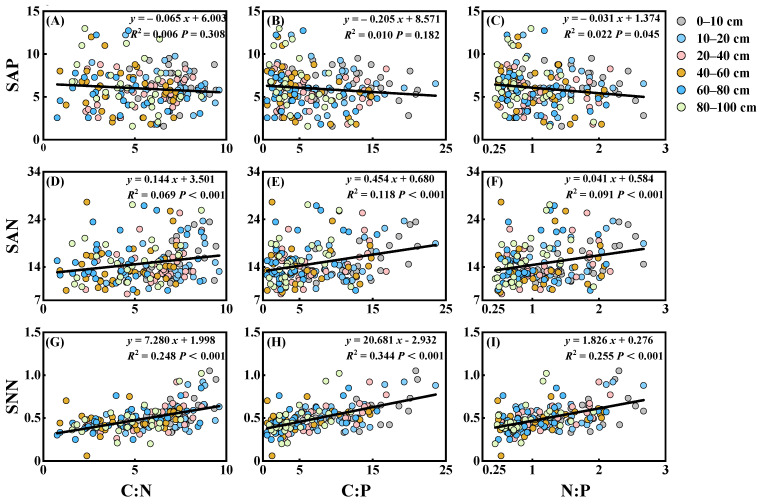
Relationship between SAP, SAN, ANN, and soil stoichiometric ratios of C, N, and P. C:N, soil carbon–nitrogen ratio; C:P, soil carbon–phosphorus ratio; N:P, soil nitrogen–phosphorus ratio; SAP, soil available phosphorus; SAN, soil ammonium nitrogen; SNN, soil nitrate nitrogen; (**A**), relationship between soil C:N ratio and SAP; (**B**), relationship between soil C:P ratio and SAP; (**C**), relationship between soil N:P ratio and SAP; (**D**), relationship between soil C:N ratio and SAN; (**E**), relationship between soil C:P ratio and SAN; (**F**), relationship between soil N:P ratio and SAN; (**G**), relationship between soil C:N ratio and SNN; (**H**), relationship between soil C:P ratio and SNN; (**I**), relationship between soil N:P ratio and SNN.

In order to further determine the relationship between the soil C, N, and P stoichiometric ratios and nutrient availability, a linear regression analysis was conducted (Figure 5). The results showed that SAP tended to decrease significantly with the increase in soil N:P ratio. The soil SAP decreased by 0.031 with an increase of 1 in the soil N:P ratio. There was a significant linear regression relationship between SAN, SNN, and the stoichiometric ratios of soil C, N, and P (*p* < 0.001). Both soil SAN and SNN tended to increase significantly with increasing soil C:N, C:P, and N:P ratios (Figure 5). An increase of 1 in the soil C:N, C:P, and N:P ratios resulted in an increase of 0.144, 0.454, and 0.041 in SAN and 7.280, 20.681, and 1.826 in SNN, respectively. This indicates that the SNN was significantly influenced by the soil C, N, and P stoichiometric ratios. In brief, the soil C, N, and P stoichiometric ratios all significantly affected the soil N and P availability.

### 2.6. The Relationship of Soil C, N, and P Stoichiometric Ratios and Plant Diversity

**Figure 6:**
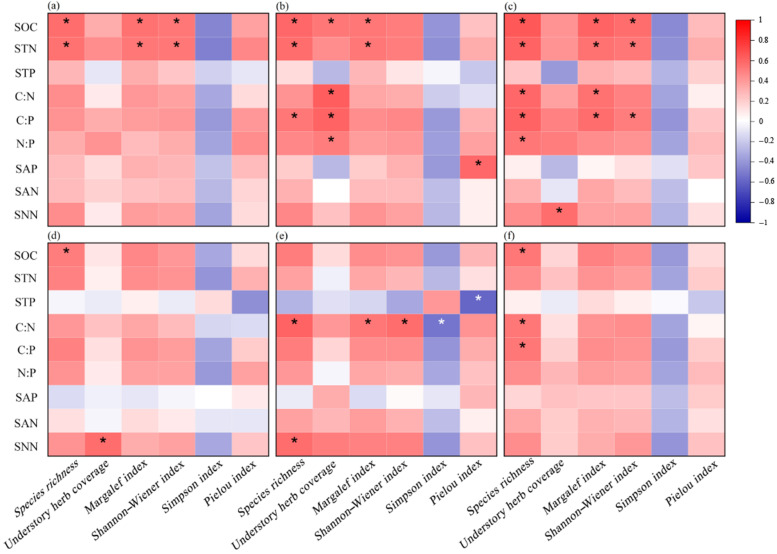
Correlation analysis of soil C, N, and P stoichiometric ratios with plant species diversity. *, *p* < 0.05; SOC, soil organic carbon; STN, soil total nitrogen; STP, soil total phosphorus; C:N, soil carbon–nitrogen ratio; C:P, soil carbon–phosphorus ratio; N:P, soil nitrogen–phosphorus ratio; SAP, soil available phosphorus; SAN, soil ammonium nitrogen; SNN, soil nitrate nitrogen; (**a**–**f**) represent 0–10 cm, 10–20 cm, 20–40 cm, 40–60 cm, 60–80 cm, and 80–100 cm soil layers, respectively.

The results depicted in Figure 6 demonstrate that the SOC and STN were significantly positively correlated with species richness, the Margalef index, and the Shannon index across the 0–10 cm, 10–20 cm, and 20–40 cm soil layers. In the 10–20 cm soil layer, there were significant positive correlations between the ratios of soil C:N, C:P, and N:P and the understory herb coverage. Additionally, a significant positive correlation was found between the C:P ratio and species richness. Within the 20–40 cm soil layer, the ratio of C:N, C:P, and N:P showed significant positive correlation with species richness. Moreover, the C:N ratio also exhibited significant positive correlation with the Margalef index, while the C:P ratio demonstrated significant positive correlation with both the Shannon index and Margalef index.

## 3. Materials and Methods

### 3.1. Study Area

The study area is located in Xining City, Qinghai Province (100°54′~101°55′ E, 36°13′~37°25′ N), at an altitude ranging from 2250 m to 3100 m and with a slope ranging from 0° to 32°. The study area has a semi-arid plateau continental climate, with an average annual temperature of 5.8 °C. The average annual precipitation is 368 mm, the rainy season is mainly from July to September, and the annual evaporation is 1100 mm, while the annual sunshine hours amount to 2600 h. Soil types in the study area are mainly chestnut soil and sierozem.

The natural vegetation of the study area is grassland, dominated by plants of the *Asteraceae*, *Poaceae*, *Leguminosae*, and *Rosaceae* families. Since 1989, this region has been continuously reforested on a large scale, with several species of trees such as *Pinus tabulaeformis* Carr, *Populus hopeiensis* Hu et Chow, and *Picea crassifolia* Kom, etc., and major shrubs such as *Caragana korshinskii* Kom, *Hippophae rhamnoides* Linn, and *Tamarix chinensis* Lour, etc.

### 3.2. Soil Sample Collection and Plant Species Diversity Calculation

**Figure 7:**
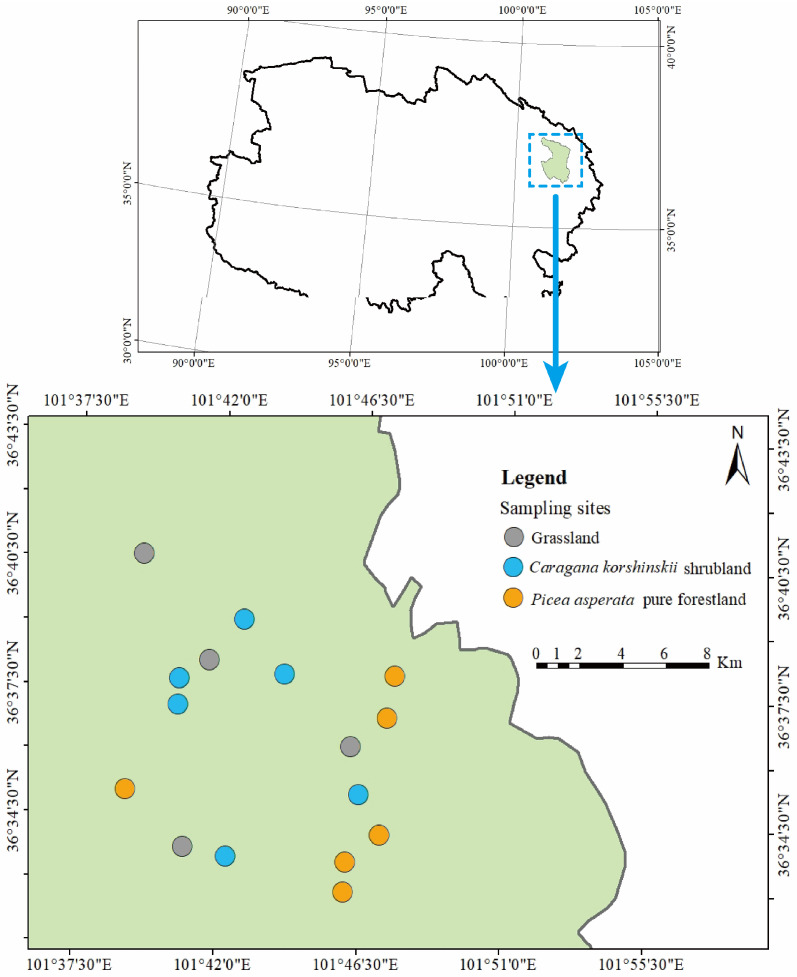
Distribution of plots in the study area.

A field survey was conducted in August 2021. Three typical vegetation types were selected, including *Picea asperata* pure forestland, *Caragana korshinskii* shrubland, and grassland with afforestation of 40a, 25a, and 10a in the north and south mountains of Xining City, as research objects. According to vegetation types and afforestation years, we ultimately selected 16 plots as sampling points, including 4 grassland plots, 6 *Caragana korshinskii* shrubland plots, and 6 *Picea asperata* pure forestland plots. The locations and basic conditions of the sampling plots are depicted in Figure 7 and Table S1. Furthermore, within each plot, three subplots were laid out along the length of the slope: a *Picea asperata* pure forestland with 20 m × 20 m size specification, a *Caragana korshinskii* shrubland with 2 m × 2 m, and a grassland with 1 m × 1 m (with intervals of greater than 10 m between subplots). Within each subplot, ten sampling points were selected according to the ‘checkerboard’ sampling method, and soil samples were taken from six levels of 0–10 cm, 10–20 cm, 20–40 cm, 40–60 cm, 60–80 cm, and 80–100 cm with a soil auger with a diameter of 3 cm. Then the soil in the same layer was mixed into a soil sample. After the collection, those samples were stored in a sealed bag and brought back to the laboratory. Plant roots, stones, and other impurities were removed from the soil samples, and then we processed the soil sample by air-drying. Finally, an appropriate amount of soil was taken using the tetrad method and then sieved through a 2 mm mesh sieve. Soil samples were stored for the determination of soil properties.

For all arbors within the subplot, the diameter at breast height (DBH), tree height, and branch height were measured using DBH calipers and Vertex ultrasonic height rangefinders (Häglof, Torsång, Sweden), while the crown width was measured using a tape measure. In the shrub layer, we investigated the ground diameter, plant height, species number, plant number, and crown width of all shrubs in the subplot. To collect data related to the herbaceous layer, we examined the species number, plant number, coverage, and weight of each species in both the understory herbaceous layer and herbaceous sample plots by setting up 1 m × 1 m herbaceous-layer sample plots. The coverage of the herbaceous plot was assessed along its diagonal. The species diversity index is a numerical measure used to assess the level and spatial distribution characteristics of species diversity. In recent years, the species richness index, species diversity index, and evenness index have been utilized in studying the diversity of plant community species. The Shannon–Wiener index, Simpson index, Margalef index, and Pielou index were utilized to characterize the species diversity of plant communities in the study area. The biodiversity index is calculated as follows:

(1) $$\mathrm{Shannon}\text{–}\mathrm{Wiener}\;\mathrm{index}:\;H^{\prime }=-\sum_{i=1}^{s}P_{i}\log_{2}P_{i}$$

(2) $$\mathrm{Simpson}\;\mathrm{index}:\;D=1-\sum P_{i}^{2}$$

(3) $$\mathrm{Margalef}\;\mathrm{index}:\;D=\frac{S-1}{\log_{2}N}$$

(4) $$\mathrm{Pielou}\;\mathrm{index}:\;J=\frac{H^{\prime }}{\log_{2}S}$$

where *S* is the total number of species, *N* is the total number of individuals of the species, and *P_i_* is the ratio of individuals belonging to species *i* to all individuals.

The plant diversity index of various places is shown in Table S2.

### 3.3. The Determination of Soil Properties

In this study, the soil organic C (SOC) concentration was measured using the acid-dichromate FeSO_4_ titration technique modified by Walkley–Black method [25]. After digestion with H_2_SO_4_ and H_2_O_2_, the Kjeldahl method was used to determine the concentrations of soil total nitrogen (STN). The concentrations of soil total phosphorus (STP) were determined using the colorimetric method with molybdenum antimony reagent. The soil available phosphorus (SAP) was measured using the Olsen method [26]. The soil ammonium nitrogen (SAN) and nitrate nitrogen (SNN) were measured using a continuous flow analyzer (Auto Analyzer 3, SEAL, Hamburg, Germany). The soil pH was determined via 1:2.5 soil-to-water ratio [26]. We further determined soil water concentrations (SWC), soil total porosity (Ps), and soil bulk density (BD) by constant head method, and the determination method was referred to as <<Determination of Forest Soil Moisture—Physical Properties>> (LY/T1215-1999) [27].

### 3.4. Statistical Analysis

Soil stoichiometric ratios (C:N, C:P, and N:P) were calculated by the ratio of SOC to STN, the ratio of SOC to STP, and the ratio of STN to STP. The data were collated using Microsoft Excel 2019. ANOVA and Pearson correlation analyses were conducted using IBM SPSS Statistics 27 (SPSS, Inc., Chicago, IL, USA). Principal component analyses (PCA) were performed with Canoco 5. All data in the figures and tables are expressed as mean ± standard error. Before conducting statistical analyses, all data were transformed using the log(x + 1) function to ensure a normal distribution (*p* > 0.05). The concentrations and ratios of SOC, STN, and STP in the same soil layer at different restoration times, as well as in different soil layers at the same restoration time, were analyzed using one-way ANOVA. The significance of the data was assessed using Duncan’s test (*p* < 0.05). The effects of restoration time, vegetation type, and soil depth on soil concentrations of SOC, STN, STP, SAP, SAN, and SNN as well as C:N, C:P, and N:P ratios were examined using multifactor ANOVA. Pearson’s method was employed to investigate the relationship between soil C, N, and P stoichiometric ratios and soil physicochemical properties as well as plant species diversity. Principal component analysis (PCA) was performed using Canoco 5 to examine the distribution characteristics of soil C, N, and P stoichiometric ratios in each soil layer.

## 4. Discussion

### 4.1. Effects of Reforestation Type and Reforestation Age on Soil C, N, and P Stoichiometric Ratios

Our study characterized the soil C, N, and P stoichiometric ratios of grassland, shrubland, and forestland across reforestation times of 10a, 25a, and 40a. In general, the mean values of soil C:N, C:P, and N:P ratios in this study were lower than the average values of these ratios in China (11.9, 52.7, and 3.9) [28]. This indicates that the decomposition and mineralization rates of the soil organic matter in the study area are notably high, resulting in a high potential for organic matter decomposition and P release. Consequently, vegetation growth is severely limited by N in this region.

The variations in soil stoichiometric ratios of C, N, and P exhibited divergent trends across grassland, shrubland, and arborland during the process of reforestation. These results are inconsistent with both the findings of previous studies and Hypothesis (i). Guo et al. [29], in the southwest karst region of China, found changes in the soil C:N ratio listed in descending order as follows: subtropical forestland, sparse forestland, shrub forestland, grassland. As for the soil C:P ratio, the descending order became as follows: sparse forestland, subtropical forestland, shrub forestland, grassland. For the soil N:P ratio, it was sparse forestland, shrub forestland, subtropical forestland, grassland. The variations in soil elemental stoichiometric ratios among different vegetation types primarily arise from diverse inputs of exogenous materials. In our study, compared to grassland, the 25a of arbor reforestation and shrub reforestation had decreased soil C:N, C:P, and N:P. This is because at 25a of reforestation, both arbor reforestation and shrub reforestation significantly reduced SOC and STN, whereas they had no significant effect on STP (Table S3), and the change rate of SOC was higher than that of STN. This phenomenon indicated that soil provided a carbon and nitrogen source for 25a shrub and arbor forest in this region. Conversely, grasslands can produce highly decomposable material due to their short life cycle, weaker resilience, and the high turnover rate of the root system, resulting in higher SOC and STN [30,31]. At 40a of reforestation, compared to grassland, shrub reforestation enhanced soil C:N, C:P, and N:P ratios, while arbor reforestation only reduced the C:N ratio and enhanced the N:P ratio of the surface soil. Accordingly, we found that compared to grassland, shrub reforestation had significantly increased SOC and STN, whereas it made no significant difference on STP; moreover, the change rate of SOC was higher than that of STN (Table S3). The higher SOC and STN in shrubland may be attributed to more litter and roots (Table S1). Firstly, the above-ground biomass of shrubland increased (Table S1), which in turn resulted in increased carbon inputs of above-ground litter to shrub reforestation. Secondly, roots of shrubs gradually became longer and thicker with the increase of reforestation time, and more root residues and rhizodeposition were produced. In addition, arbor reforestation had no significant influence on SOC due to its coniferous and stand structure [32]. STN concentrations of surface soil showed an increase with prolonged afforestation [33,34], whereas STP of the surface soil was reduced (Table S3). This indicates that *Picea asperata* pure forestland exhibits greater phosphorus utilization in the later stages of afforestation. Furthermore, our study area is located in the typical alpine–cold arid region in China, which could explain the inconsistency of our results with those of others.

The soil C:N, C:P, and N:P ratios exhibited distinct temporal trends in response to reforestation in the grassland, *Caragana korshinskii* shrubland, and *Picea asperata* pure forestland. Specifically, the 25a grassland displayed higher ratios compared to both the 40a and 10a grasslands. In the *Caragana korshinskii* shrubland, the ratios followed a decreasing order of 40a, 25a, and 10a. However, no significant difference was observed among these in the *Picea asperata* pure forestland. Therefore, the results of *Caragana korshinskii* shrubland are consistent with our Hypothesis (ii), whereas the results of grassland and *Picea asperata* pure forestland are inconsistent with our Hypothesis (ii). The decrease in the stoichiometric ratio of the grassland after 40a may be attributed to grassland degradation, which results in a reduction in the inputs of soil organic matter, ultimately affecting the accumulation of SOC and STN [35,36]. The findings of this study on *Caragana korshinskii* shrubland are in line with those reported by Pan et al. [37], who found a similar increasing trend in the external soil C:P ratio and N:P ratio with increasing stand age for different planting years (9a, 17a, 27a) of scrub (planted *Caragana korshinskii* shrubland) in desert grassland. The results for the *Picea asperata* pure forestland in our study diverged from those reported by Deng et al. [38], who investigated the leaf, soil, and microbial carbon, nitrogen, and phosphorus stoichiometry of *Robinia pseucdoacacia* (at 5a, 10a, 20a, 30a, and 45a) in the loess hilly area. Their findings showed that the soil C:N ratios decreased (at 5a–20a) and then increased (at 20a–45a) with restoration time, and that both soil C:P and N:P ratios increased with restoration time. The difference may be due to the different tree species. The slower decomposition rate of litter material in *Picea asperata* pure forestland, due to its coniferous nature, results in a slower supply of various nutrients to the soil compared to Robinia pseudoacacia forestland [39]. Consequently, there were no significant differences observed in the soil carbon, nitrogen, and phosphorus stoichiometric ratios at 10a, 25a, and 40a for *Picea asperata* pure forestland.

The stoichiometric ratios of soil carbon, nitrogen, and phosphorus in each vegetation type showed a decreasing trend with soil depth at different restoration times. These findings are consistent with the results of Ren et al. [40] regarding the chemometric characteristics of acacia soils of different ages on the Loess Plateau. In the process of vegetation restoration, SOC and STN initially accumulate in the soil surface layer, and then gradually migrate downward and diffuse due to leaching, forming a distribution pattern that gradually decreases with increasing soil depth [41]. In contrast, soil P is categorized as a sedimentary element, which is mainly produced through gradual mineral weathering, and so the source is relatively stable [42], resulting in small spatial variations in soil P and no significant difference with increasing soil depth [43]. It is worth noting that the soil C:N, C:P, and N:P ratios of shrubland were significantly higher than those of arborland and grassland in the 40–100 cm soil layer. This indicates that in our study area, long-term shrub afforestation could improve the accumulation of SOC and STN in the deep soil layer.

### 4.2. Soil C, N, and P Stoichiometric Ratios Significantly Influence Nutrient Availability

The results of the regression analysis revealed a strong correlation between the stoichiometric ratios of soil C, N, and P and nutrient availability. The soil SAP showed a significant negative correlation with soil N:P ratios, with an *R*^2^ of 0.022 (*p* < 0.05). Additionally, the soil SAN exhibited significant positive correlations with the C:N, C:P, and N:P ratios, with *R*^2^ values of 0.069 (*p* < 0.001), 0.118 (*p* < 0.001), and 0.091 (*p* < 0.001), respectively. Similarly, the soil SNN displayed significant positive correlations with the C:N, C:P, and N:P ratios, with *R*^2^ values of 0.248 (*p* < 0.001), 0.344 (*p* < 0.001), and 0.255 (*p* < 0.001), respectively. These findings contradicted some existing studies. For example, Ma et al. [44], in their study of soil stoichiometric ratios of forestland soils at different altitudes in the Qinling Mountains, showed that soil SAN and SNN in *Quercus aliena var. acuteserrata* forestland, *Quercus liaotungensis* forestland, and *Betula albo-sinensis var. septen-trionalis* forestland showed a negative correlation with soil C:N ratios (>12); and Gao et al. [45], in their study of the effect of fallowing in the Ningnan mountainous area on the nitrogen composition of the soil, showed that soil SAN and SNN were negatively correlated with soil C:N ratios (10.91–23.30). Chu et al. [46] showed a negative correlation between soil SAN and soil C:N ratios (17.9–31.0) for the nutrient availability and nitrogen mineralization potential of boreal tundra soils. Gundersen et al.’s [47] research on forest ecosystems showed that SNN was negatively correlated with soil C:N ratio. For the present study, the soil C, N, and P stoichiometric ratios significantly influenced soil SNN, and the soil N:P ratio significantly influenced the SAP. Therefore, we deduced that there may be a critical value for the influence of soil C:N ratio on soil available nitrogen. When the soil C:N ratio is greater than this critical value, the soil C:N ratio is significantly negatively correlated with soil available nitrogen, and when the soil C:N ratio is less than this critical value, the soil C:N ratio is significantly positively correlated with soil available nitrogen. In addition, several models of nitrogen saturation incorporate a breakthrough function, whereby nitrogen begins to be leached at C:N ratios below an upper threshold and is completely leached at ratios below a lower threshold [48]. Rowe et al. [48] demonstrated that different vegetation communities had different C:N ratio thresholds for soil nitrogen saturation or leaching. In our study, the significant positive correlation of SAN and SNN with the C:N ratio (<10) may be due to the ‘digging nitrogen’ effect of K-strategist microbial taxa. Compared to grassland, afforestation alters litter inputs and creates a carbon-rich and nitrogen-rich environment in the upper soil layers, which contributes to the development of fast-growing r-strategist microbial taxa [49]. Nitrogen depletion via microbial growth leads to lower soil nitrogen concentration, and nitrogen depletion is further exacerbated by the easy-to-utilize carbon that is secreted into the soil through the plant root system. Therefore, subsoil microorganisms are likely to be dominated by the slower-growing K-strategist microbial taxa, and are required to obtain nitrogen through the decomposition of recalcitrant organic matter in order to maintain a balance in the elemental composition of their own biomass [50,51,52]. Overall, the relationship between the soil C:N ratio and soil available nitrogen is still uncertain, and thus the potential mechanism needs to be further explored.

### 4.3. Plant Diversity Affected Soil C, N, and P Stoichiometric Ratios in Upper Soil Layer

Consistent with our Hypothesis (iii), we found that the stoichiometric ratios of SOC, STN, and STP in the upper soil layer were significantly affected by the plant diversity and the plant species richness. In our study, the change in stoichiometric ratios is a result of the change in SOC and STN. There are several mechanisms that may lead to higher SOC and STN through plant diversity. On the one hand, increased plant species richness and diversity can indirectly enhance SOC storage through positive impacts on ecosystem productivity [53]. On the other hand, high plant diversity would increase root input, thereby promoting carbon uptake by rhizosphere microorganisms and resulting in an increase in microbial necromass over time. Ultimately, this indirectly promotes the accumulation of SOC [54]. Therefore, plant diversity and plant species richness affect SOC via the above ways and further affect the stoichiometric ratios of SOC, STN, and STP in the upper soil layer.

## 5. Conclusions

In this paper, the ecological stoichiometric characteristics of soil carbon, nitrogen, and phosphorus in the 0–100 cm soil layer were analyzed at different restoration times in *Picea asperata* pure forestland, *Caragana korshinskii* shrubland, and grassland in the alpine–cold arid region on the Loess Plateau, China. We found that compared to grassland, both *Picea asperata* reforestation and *Caragana korshinskii* reforestation showed a decrease in soil C:N, C:P, and N:P ratios at 25a of reforestation. However, at 40a of reforestation, *Caragana korshinskii* reforestation increased soil C:N, C:P, and N:P ratios, and *Picea asperata* reforestation lowered the C:N ratio, at the same time making the N:P ratio higher on the surface soil. In addition, the soil C:N, C:P, and N:P ratios of the three vegetation types showed different trends with the increase of restoration time. The ratios were higher in the 25a grassland compared to the 40a and 10a grasslands. In *Caragana korshinskii* shrubland, the ratios decreased in the order of 40a followed by 25a and then 10a. There were no significant differences observed in *Picea asperata* pure forestland. Meanwhile, only the C:N, C:P, and N:P ratios in the deep soil (40–100 cm) of *Caragana korshinskii* shrubland increased with afforestation time. The available phosphorus in soil will decrease as the soil N:P ratio increases, while ammonium nitrogen and nitrate nitrogen will increase with higher soil C:N, C:P, and N:P ratios. Plant diversity affected soil C, N, and P stoichiometric ratios in the upper soil layer. However, parameters such as plant C, N, and P; plant root biomass; and soil microbial biomass were not considered in this study. If these parameters were incorporated, it would establish a comprehensive plant–microbial–soil continuum that better reflects the impact of afforestation on the soil–plant ecosystem. Overall, this study provides valuable insights into the impacts of reforestation on soil nutrients and the selection of reforestation species in the alpine–cold arid region of Loess Plateau, China. Specifically, the *Caragana korshinskii* shrubland enhances the availability of N and P by adjusting the soil element stoichiometric ratio, thereby ensuring long-term co-development between plants and soil in this region.

## Figures and Tables

**Table 1:** Three-factor variance analysis of soil element concentrations and stoichiometric ratios (F value).

Factor	SOC	STN	STP	SAP	SAN	SNN	C:N	C:P	N:P
Restoration time	26.486 ***	27.223 ***	15.993 ***	1.284	6.206 **	22.666 ***	3.9000 *	30.487 ***	28.726 ***
Vegetation type	26.163 ***	15.411 ***	1.108	0.431	2.087	23.078 ***	22.005 ***	23.359 ***	11.84 ***
Soil depth	38.792 ***	32.527 ***	0.736	0.755	1.054	2.378 *	14.688 ***	33.352 ***	22.704 ***
Restoration time × Vegetation type	26.992 ***	28.071 ***	2.129	5.185 ***	11.491 ***	5.096 ***	6.095 ***	22.641 ***	20.032 ***
Restoration time × Soil depth	2.806 **	2.278 *	0.194	1.008	1.285	0.935	0.884	2.919 **	1.987 *
Vegetation type × Soil depth	1.078	0.900	0.461	0.852	0.883	0.729	0.939	0.889	0.556
Restoration time × Vegetation type × Soil depth	1.054	0.824	0.74	0.555	0.864	0.790	0.839	0.711	0.619

Notes: *, *p* < 0.05; **, *p* < 0.01; ***, *p* < 0.001.

**Table 2:** Correlation analysis between soil stoichiometric ratio and physico-chemical properties.

	SAP	SAN	SNN	BD	P_S_	pH
Soil C:N	−0.103	0.157 *	0.474 ***	−0.425 ***	0.274 ***	−0.152 *
Soil C:P	−0.063	0.227 **	0.486 ***	−0.539 ***	0.339 ***	−0.217 **
Soil N:P	−0.085	0.218 **	0.406 ***	−0.527 ***	0.333 ***	−0.229 **

Notes: *, *p* < 0.05; **, *p* < 0.01; ***, *p* < 0.001; SAP, soil effective phosphorus; SAN, soil ammonium nitrogen; SNN, soil nitrate nitrogen; BD, soil bulk density; Ps, soil total porosity; pH, soil pH.

## Data Availability

The data presented in this study are available on request from the corresponding author.

## Supplementary Materials

The following supporting information can be downloaded at: https://www.mdpi.com/article/10.3390/plants13162320/s1, Table S1: The information of sampling site in the study area; Table S2: The plant species diversity index; Table S3: Characteristics of soil C, N, and P concentrations in different restoration years and vegetation types.
