# Supplementary material for: The Critical Role of Soil Ecological Stoichiometric Ratios: How Does Reforestation Improve Soil Nitrogen and Phosphorus Availability?
Source: Plants (Basel). 2024 Aug 20;13(16):2320. doi: 10.3390/plants13162320 (PMC11358991; doi:10.3390/plants13162320)
Supplement: Supplementary file 1 [file plants-13-02320-s001.zip › plants-3119133-supplementary.pdf]

Supplementary materials

Table S1. The information of sampling site in the study area.

Sites	Restoration ages/a	Vegetation types	Longitude	Latitude	Altitude/ m	Gradients/ °	Slope	Dominant plant	DBH/ cm	Height/ m	Biomass t/hm^2				
											Aboveground part	Root	Whole plant	Understory herb aboveground part	Understory herb underground part
1	40	grassland	101°41′35″E	36°38′08″N	2389	25.0	Shade slope	*Artemisia capillaris*、*Heteropappus hispidus*、*Tribulus terrester*	/	/	1.32	6.28	7.60	/	/
2	25	grassland	101°46′08″E	36°36′14″N	2564	29.0	Shade slope	*Medicago archiducis-nicolai*、*Geranium wilfordii*、*Medicago falcata*	/	/	1.52	7.24	8.76	/	/
3	10	grassland	101°39′27″E	36°40′35″N	2750	37.8	Shade slope	*Agropyron cristatum*、*Heteropappus hispidus*、*Conyza canadensis*	/	/	1.99	9.47	11.45	/	/
4	10	grassland	101°40′56″E	36°33′46″N	2619	14.6	Shade slope	*Agropyron cristatum*、*Heteropappus hispidus*、*Medicago archiducis-nicolai*	/	/	2.29	10.91	13.21	/	/
5	40	*Caragana korshinskii*	101°40′39″E	36°37′41″N	2537	10.0	Sunny slope	*Caragana korshinskii*	/	/	12.71	4.68	17.39	1.99	9.46
6	40	*Caragana korshinskii*	101°40′40″E	36°37′41″N	2526	18.0	Sunny slope	*Caragana korshinskii*	/	/	8.12	3.02	11.14	3.42	16.27
7	25	*Caragana korshinskii*	101°46′26″E	36°35′07″N	2434	37.2	Shade slope	*Caragana korshinskii*	/	/	3.06	1.25	4.31	1.97	9.38
8	10	*Caragana korshinskii*	101°42′40″E	36°39′08″N	2607	31.9	Shade slope	*Caragana korshinskii*	/	/	2.64	1.10	3.74	1.19	5.68
9	10	*Caragana korshinskii*	101°43′18″E	36°33′34″N	2568	32.0	Half shade slope	*Caragana korshinskii*	/	/	8.85	3.27	12.12	3.28	15.63
10	10	*Caragana korshinskii*	101°44′00″E	36°37′53″N	2791	8.2	Shade slope	*Caragana korshinskii*	/	/	4.41	1.61	6.02	5.48	26.10
11	40	*Picea asperata* pure forestland	101°47′28″E	36°37′55″N	2343	34.9	Sunny slope	*Picea asperata*	4.74	3.60	16.90	31.42	48.32	2.00	9.51
12	25	*Picea asperata* pure forestland	101°46′02″E	36°33′25″N	2617	26.6	Half sunny slope	*Picea asperata*	4.69	2.05	3.85	11.43	15.29	3.09	14.74
13	25	*Picea asperata* pure forestland	101°46′04″E	36°33′32″N	2630	19.4	Half shade slope	*Picea asperata*	5.25	2.05	5.35	12.28	17.62	1.64	7.80
14	25	*Picea asperata* pure forestland	101°47′15″E	36°36′55″N	2480	30.1	Half shade slope	*Picea asperata*	4.54	2.61	6.80	12.28	19.08	1.61	7.65
15	25	*Picea asperata* pure forestland	101°47′07″E	36°34′11″N	2560	3.3	Sunny slope	*Picea asperata*	6.08	3.49	14.47	20.20	34.67	2.92	13.92
16	10	*Picea asperata* pure forestland	101°39′05″E	36°35′04″N	2615	21.2	Shade slope	*Picea asperata*	3.40	1.32	1.33	5.83	7.16	1.85	8.79

Table S2. The plant species diversity index.

Sites	Species number	Understory herb coverage	Margalef index	Shannon-Wiener index	Simpson index	Pielou index
1	8	0.6333	1.2439	1.8593	0.1800	0.9136
2	12	0.9000	2.1336	2.3551	0.1066	0.9503
3	11	0.7333	1.9138	2.1906	0.1291	0.9155
4	/	/	/	/	/	/
5	9	0.4833	1.5816	1.8769	0.1872	0.8734
6	10	0.7500	1.6128	2.0178	0.1558	0.8924
7	8	0.9500	1.2851	1.9473	0.1541	0.9626
8	10	0.6667	1.6720	2.1350	0.1377	0.9306
9	5	0.5833	0.7281	1.3756	0.2848	0.9214
10	3	0.7667	0.5154	0.7777	0.5625	0.6573
11	3	0.5333	0.5814	0.9321	0.4455	0.8533
12	6	0.7600	0.9475	1.4632	0.2773	0.8969
13	9	0.6800	1.6800	1.8958	0.2039	0.8617
14	7	0.7167	1.3914	1.6313	0.2474	0.8400
15	8	0.7367	1.5550	1.7997	0.2126	0.8655
16	7	0.4875	1.3486	1.7362	0.2115	0.9030

Table S3. Characteristics of soil C, N, and P concentrations in different restoration years and vegetation types.

Element	Vegetation types	Restoration ages/a	Soil depth/(cm) 0-10	10-20	20-40	40-60	60-80	80-100
SOC	grassland	40	9.38±0.49βBa	4.36±0.68βBb	2.35±0.60βBc	1.54±0.40βBc	1.81±0.22βBc	2.35±0.52βAc
		25	20.58±0.59αAa	18.62±0.46αAab	13.46±4.18αAbc	8.82±1.24αAcd	7.52±0.59αAcd	4.45±1.18αAd
		10	7.48±0.93αBa	7.92±1.77αBa	6.69±1.59αBa	4.91±2.10αABa	4.75±1.50αABa	3.49±1.06αAa
	Caragana korshinskii	40	14.94±1.38αAa	12.16±1.49αAab	10.32±0.49αAb	8.61±1.12αAbc	6.33±1.77αAc	5.30±0.97αAc
		25	10.59±2.36βABa	11.63±6.14βAa	8.69±4.38αβAa	2.68±1.24βBa	3.14±0.40αABa	2.55±0.98αBa
		10	7.69±1.17αBa	7.00±1.00αAa	3.63±0.54αβBb	2.79±0.35αBb	1.77±0.46αBb	2.26±0.35αBb
	Picea asperata pure forestland	40	9.38±1.34βAa	4.22±2.35βAb	2.62±1.41βAb	1.41±1.01βAb	0.81±0.00βAb	1.01±0.34βAb
		25	9.93±1.22βAa	7.17±0.81βAa	4.13±1.17βAb	3.01±0.76βAb	3.02±1.02αAb	3.54±1.08αAb
		10	6.02±3.01αAa	3.53±1.83αAa	2.81±0.46βAa	2.75±1.44αAa	2.16±0.33αAa	1.90±0.33αAa
STN	grassland	40	1.10±0.05βBa	0.62±0.08βCb	0.44±0.04γBc	0.41±0.06βBc	0.39±0.04βBc	0.35±0.02βBc
		25	2.35±0.03αAa	2.13±0.08αAab	1.66±0.35αAbc	1.26±0.20αAcd	0.93±0.01αAd	0.83±0.07αAd
		10	1.09±0.09αBab	1.17±0.11αBa	0.91±0.16αBab	0.82±0.23αABab	0.68±0.15αABb	0.62±0.10αABb
	Caragana korshinskii	40	1.68±0.16αAa	1.49±0.21αAab	1.33±0.03αAabc	1.19±0.14αAbc	1.08±0.18αAbc	0.90±0.07αAc
		25	1.64±0.47βAa	1.35±0.64αβAa	1.15±0.53αβABa	0.69±0.14βBa	0.59±0.04αBa	0.67±0.03αBa
		10	1.10±0.14αAa	0.97±0.09αβAa	0.70±0.03αBb	0.64±0.08αBb	0.61±0.06αBb	0.48±0.02αCb
	Picea asperata pure forestland	40	1.30±0.13αβAa	0.81±0.37βAab	0.62±0.12βAb	0.51±0.07βAb	0.48±0.02βAb	0.43±0.04βAb
		25	1.32±0.10βAa	1.10±0.13βAa	0.71±0.07βAb	0.69±0.08βAb	0.70±0.11αAb	0.74±0.13αAb
		10	0.88±0.24αAa	0.69±0.05βAa	0.60±0.02αAa	0.69±0.14αAa	0.62±0.05αAa	0.51±0.05αAa
STP	grassland	40	0.69±0.02αβAa	0.63±0.03αAab	0.66±0.02αAab	0.62±0.02αAb	0.65±0.01Bαab	0.69±0.02αABab
		25	0.82±0.04αAa	0.75±0.02αAab	0.68±0.03αAbc	0.68±0.03αAbc	0.66±0.03αBbc	0.62±0.00αBc
		10	0.77±0.05αAa	0.77±0.06αAa	0.72±0.06αAa	0.68±0.07αAa	0.78±0.02αAa	0.77±0.04αAa
	Caragana korshinskii	40	0.76±0.04αABa	0.74±0.07αAa	0.73±0.04αAa	0.70±0.03αAa	0.63±0.04αAa	0.66±0.02αAa
		25	0.63±0.01αBa	0.64±0.03αAa	0.71±0.08αAa	0.68±0.03αAa	0.69±0.02αAa	0.71±0.01αAa
		10	0.84±0.05αAa	0.75±0.02αAab	0.70±0.05αAb	0.77±0.04αAab	0.76±0.04αAab	0.69±0.02αAb
	Picea asperata pure forestland	40	0.62±0.00βAa	0.65±0.02αAa	0.68±0.08αAa	0.61±0.06αAa	0.67±0.04αAa	0.63±0.03αAa
		25	0.77±0.05αAa	0.74±0.05αAa	0.70±0.04αAa	0.71±0.04αAa	0.74±0.02αAa	0.75±0.05αAa
		10	0.77±0.02αAa	0.83±0.02αAa	0.87±0.07αAa	0.82±0.22αAa	0.85±0.21αAa	0.76±0.10αAa

Notes: Greek letters indicate significant differences among different vegetation types with the same reforestation time ($p < 0.05$), capital letters indicate significant differences among different reforestation time under the same vegetation types ($p < 0.05$), and lowercase letters indicate significant differences among different soil depths in the same year ($p < 0.05$).
